# Prediction of university fund revenue and expenditure based on fuzzy time series with a periodic factor

**DOI:** 10.1371/journal.pone.0286325

**Published:** 2023-05-25

**Authors:** Yueqian Shen, Xiaoxia Ma, Yajing Sun, Sheng Du

**Affiliations:** 1 Department of Finance and Asset Management, China University of Geosciences, Wuhan, China; 2 Audit Department, China University of Geosciences, Wuhan, China; 3 School of Automation, China University of Geosciences, Wuhan, China; 4 Hubei Key Laboratory of Advanced Control and Intelligent Automation for Complex Systems, Wuhan, China; 5 Shenzhen ET Technology Co., Ltd, Shenzhen, China; National University of Sciences and Technology, PAKISTAN

## Abstract

Financial management and decision-making of universities play an essential role in their development. Predicting fund revenue and expenditure of universities can provide a necessary basis for funds risk prevention. For the lack of solid data reference for financial management and funds risk prevention in colleges and universities, this paper presents a prediction model of University fund revenue and expenditure based on fuzzy time series with a periodic factor. Combined with the fuzzy time series, this prediction method introduces the periodic factor of university funds. The periodic factor is used to adjust the proportion of the predicted value of the fuzzy time series and the periodic observation value. A fund revenue prediction model and a fund expenditure prediction model are constructed, and an experiment is carried out with the actual financial data of a university in China. The experimental result shows the effectiveness of the proposed model, which can provide solid references for financial management and funds risk prevention in universities.

## 1. Introduction

In recent years, China’s higher education has developed rapidly, the scale of enrollment in universities has gradually expanded, and the number of open-source channels has become more and more numerous. Besides, the total amount of funds has also increased, which has put forward higher and clearer requirements for managing funds in universities. According to the data of the <National Bureau of Statistics and China Education Funding Statistical Yearbook>, China’s GDP has basically stabilized at about 10% since 2017, the national economy has a stable rise in general, and the federal education funding investment has been steadily at the ratio of 4%. According to the data in the <Education Briefly> published by the OECD [1], the education investment in the member countries of the OECD accounts for GDP, the average proportion of the total is 5%, and China’s education investment is in the average world level since 2012. The investment in higher education in 2018 will be 251.1 billion yuan higher than the actual amount of investment by considering the ratio of investment in higher education to national GDP in 2012 (1.58%). This investment indicates that although the proportion of national investment in Education to GDP is increasing, the ratio of investment in higher Education to GDP is decreasing, falling to 1.51% in 2018 [2]. The growth rate of national investment in higher education is gradually slowing down, which is contradictory to universities’ rapid pace of development.

All along, universities are based on the income model of financial allocation and supplemented by other channels, so they are more dependent on the national financial policy in terms of revenue. Since 2020, due to the epidemic, the national financial input to universities has been tightened, and the rapid development rhythm makes the expenditures steadily rising challenging to fall. The continuous growth of the enrollment scale has not resulted in the same proportional growth of financial input, and the direction of funds management in universities has gradually shifted from funds deposit management to risk management. Since the 19th National Congress of the Communist Party of China, the Ministry of Finance and the Ministry of Education have issued some documents on internal control construction and funds deposit management, which put forward more clear requirements on universities’ internal control and funds security. Although universities implement national policies step by step, the mismatch between school development and national investment has caused a shortage of funds in most universities, changing the balance of school operation, "keeping the revenue to the expenditure," resulting in deficits in revenue and expenditure. "Open source and reduce expenditure, improve quality and efficiency" has become the development direction of financial management in universities.

The research on funds management in universities mainly focuses on the budget, accounting, and final account of funds, which is concerned with the process-oriented management of funds and studies on the management of funds deposits [3]. With the gradual accentuation of the funds gap problem in universities, the financial risks caused by the imbalance of fund revenue and expenditure have gradually emerged. Still, relatively little research has been conducted on the funding flow of universities. In the past, the annual financial allocation amount was fully allocated to each university after the two sessions. Still, from 2022, according to the latest requirements of the Ministry of Finance, the treasury funds are allocated according to the sequential progress, which means that the university’s funds will be used from the first half of the year, which will further highlight the contradiction of the shortage of university funds. Predicting and analyzing the revenue and expenditure of universities can prevent the financial risks and better provide more constructive opinions and suggestions for the subsequent strategic development of the university.

The role of fund revenue and expenditure prediction has become increasingly prominent, attracting many scholars. An autoregressive moving average model is used by Rahmat et al. to predict regional revenue and expenditure budget allocations in five categories of accounts [4], namely regional raw revenue, balanced funds, other legal regional revenue, indirect expenditure, and direct expenditure. Alam et al. used an artificial neural network model and an autoregressive moving average model to predict national revenue and expenditure [5]. Botrić et al. evaluated the ability of different time series models, including trend models, autoregressive moving average models, etc., to generate accurate fiscal revenue forecasts in transition countries [6] and compared them with official forecasts. The prediction effects of autoregressive models, autoregressive moving average models, and vector autoregressive models are analyzed by Streimikiene et al. Those models are used to predict direct taxes, sales taxes, excise taxes, and tariffs [7]. A hospital expense prediction model based on multivariate time series is proposed by Jing Li, which designed and developed a medical insurance big data funds prediction platform [8]. Nan Shen presented an autoregressive moving average model to perform regression fitting on the changing trends of the proportion of central fiscal revenue and the ratio of central fiscal expenditure [9].

The above methods use time-series data analysis and regression models to predict funds balances and lack of integration with emerging technologies. With the development of fuzzy theory, fuzzy time series prediction models have been well developed in forecasting, such as Taiwan Stock Exchange market capitalization weighted stock index prediction [10–19], India Bombay Stock Exchange market price prediction [20], University enrollment prediction [21, 22], urban air quality forecasting [23], energy and load forecasting [24–27], wind speed forecasting [28–31], air pollution forecasting [32–35], and so on. Therefore, the fuzzy time series prediction model has a good application prospect.

The uncertainty to be solved in this paper is the fluctuation of university fund revenue and expenditure due to fiscal policy, market environment, project schedule, etc. The fuzzy time series forecasting method is selected to solve this problem. Firstly, fuzzy methods can solve many non-linear forecasting problems by fuzzifying the data and discovering the inherent patterns between the data, so that fuzzy forecasting models have sound forecasting effects. Secondly, fuzzy methods have been fully applied in many areas, such as stock index forecasting, and market price forecasting. Besides, fuzzy methods have been fully applied in many areas, such as stock index forecasting, market price forecasting, and university enrolment forecasting, which are similar to the university fund revenue and expenditure forecasting mentioned in this paper. In conclusion, fuzzy time series forecasting methods are suitable for solving university fund revenue and expenditure forecasting.

Based on the big financial data of a university in recent years, this paper proposes a fuzzy time-series-based method for predicting fund revenue and expenditure. By introducing the periodic factor of the university fund revenue and expenditure, the periodic factor is used to adjust the fuzzy time series prediction value and the periodic observation value. The proposed method is used to construct the funds revenue prediction and expenditure prediction models, respectively. Experiments are conducted with the actual financial data of a university in China. The experimental results show the effectiveness of the proposed model, which can provide a powerful reference for the university’s financial management and financial decision-making.

## 2. Analysis of fund revenue and expenditure on a university

With the rapid development of universities and the increasing contradiction of funds shortage, accurate prediction and scientific analysis of fund revenue and expenditure are of great significance for decision-makers and funds users to make reasonable funds use strategies [36]. From the perspective of decision-makers, the prediction of fund revenue and expenditure is conducive to timely adjustment of relevant measures for the use of funds, improving the foresight of strategy formulation, achieving real-time dynamic adjustment, and matching the strategic development with the fund’s situation. Besides, it is conducive to allocating resources in a focused manner and improving the efficiency of funds use, building a whole-process funds risk prevention and control system, and incorporating fund revenue and expenditure risk prevention and control into the construction of the internal control system. From the perspective of funds users, the prediction of fund revenue and expenditure is conducive to timely adjustment of expenditure structure according to the funds dynamics of the university because some universities have implemented the approval system of many funds at each level. Avoiding the peak period of funds expenditure will effectively shorten the cycle of funds use and improve the efficiency of funds use.

Especially for teaching reform projects, many universities respond to the national policy to drive teaching reform with project research. From 2014 to 2019, the total funding of teaching reform projects in universities has increased by 184%. The number of projects has increased nearly eight times. The categories of projects have developed from a large category at the beginning to six categories now. The rapid growth of teaching reform projects has made it difficult to manage and supervise. The increase in project categories and numbers has also changed the structure of project expenditures, which has compromised project implementation effectiveness and funds use. Different project categories, research contents (subject directions), and implementation progress of teaching reform projects will lead to other expenditure structures. The prediction of fund revenue and expenditure can effectively improve the budget execution progress of teaching reform projects and strictly implement the requirements of the Ministry of Education on budget execution progress. This avoids spending money unexpectedly, arranges the progress and structure of funds expenditure more reasonably and effectively, and ensures that the expected goals can be achieved.

The total revenue of a university’s teaching reform project in 2019 reached more than 17 million, and the amount had been fully allocated before April. However, the more concentrated expenditure time of the project was mainly in June (about 4 million, accounting for 23% of the total expenditure) and September (more than 4.6 million, accounting for 27% of the total expenditure). This phenomenon coincided with the assessment nodes of the Ministry of Education on the implementation progress of special funds for each university (the implementation rate in June reached 50% and 75% in September). Those numbers also reflect a common problem of performance execution assessment of special funds in universities, which is only based on the implementation rate of funds. That is easy to cause universities to spend money unexpectedly at the assessment time point and lack efficiency in using funds. In 2020, the total funding for this university’s teaching reform project reached more than 18 million, fully allocated in May. Still, due to the inertia of project expenditure and the epidemic, the execution rate did not get the requirement in June. The higher authorities withdrew nearly 5 million yuan of the amount (which had specific epidemic reasons). Spending money unexpectedly at the assessment node does not necessarily achieve the rate of funds execution required by the Ministry of Education. There is a risk of funds being recovered. Therefore, the prediction of fund revenue and expenditure can effectively improve the budget execution progress of teaching reform projects.

Through the above analysis, the prediction of fund revenue and expenditure of universities is essential for decision-makers and funds users to make reasonable strategies. This prediction will help to adjust the relevant measures of funds use in time, focus on resource allocation, build a whole-process funds risk prevention and control system, and then improve the efficiency of funds use. To this end, this paper proposes a prediction model of university fund revenue and expenditure based on fuzzy time series with a periodic factor.

## 3 Prediction model of fund revenue and expenditure

This section first introduces the relevant definitions of fuzzy time series, then designs prediction models of university fund revenue and expenditure based on fuzzy time series with a periodic factor according to the relevant definitions, and presents the detailed steps.

### 3.1 Definition of fuzzy time series

This section will introduce the relevant definitions of fuzzy time series [37, 38], mainly including the following four definitions.

**Definition** 1: Define *U* as the universe of fuzzy time series, and divide the universe of universe *U* into finite *n* ordered subsets, which is the fuzzy interval, which can be expressed as *U* = {*u*_1_, *u*_2_, ⋯, *u*_*n*_}. Assuming that *A* is a semantic variable set on the universe *U*, that is, a fuzzy set,

$$A=\frac{f_{A}\left(u_{1}\right)}{u_{1}}+\frac{f_{A}\left(u_{2}\right)}{u_{2}}+\cdots +\frac{f_{A}\left(u_{n}\right)}{u_{n}}$$ (1)

where *f*_*A*_ is the fuzzy membership function on the fuzzy set *A*, *f*_*A*_: *U* → [0,1], and *f*_*A*_(*u*_*i*_) is the membership value of *u*_*i*_ on *A*.

**Definition** 2: Assuming that *Y*(*t*)(*t* = 1, 2, …) is a subset of real numbers, *f*_*i*_(*t*)(*t* = 1, 2, …) is defined as a set of fuzzy sets on the universe of *Y*(*t*), if *F*(*t*) contains all fuzzy sets *f*_*i*_(*t*)(*i* = 1, 2, …), namely *F*(*t*) = {*f*_1_(*t*), *f*_2_(*t*), …}, then *F*(*t*) is defined as a fuzzy time series on *Y*(*t*).

**Definition** 3: *F*(*t*) is a set of data that changes with time, if there is a relationship *R*(*t*, *t* − 1), can be expressed as,

$$F\left(t\right)=F\left(t-1\right)\circ R(t,t-1)$$ (2)

where "∘" represents a composite operation. The above formula shows that *F*(*t*) is derived from *F*(*t* − 1) with the relationship *R*(*t*, *t* − 1), then the fuzzy logic relationship between *F*(*t*) and *F*(*t* − 1) can be expressed as,

$$F\left(t-1\right)\to F\left(t\right)$$ (3)

If *F*(*t*) a fuzzy time series, let *F*(*t* − 1) = *A*_*i*_, *F*(*t*) = *A*_*j*_, then the fuzzy logic relationship between two consecutive training data *F*(*t*) and *F*(*t* − 1)can be expressed as *A*_*i*_ → *A*_*j*_.

**Definition** 4: Fuzzy logic relations with the same antecedent can form a fuzzy logic relation group. Assuming that there is a set of fuzzy logic relationships, *A*_*i*_ → *A*_*j*1_, *A*_*i*_ → *A*_*j*2_, …, the corresponding fuzzy logic relationship group is *A*_*i*_ → *A*_*j*1_, *A*_*j*2_, ⋯.

### 3.2 Prediction model based on fuzzy time series with a periodic factor

In this section, according to the definition of fuzzy time series, a fund revenue and expenditure prediction model based on fuzzy time series with a periodic factor is built (this section takes the fund expenditure prediction model as an example), which mainly includes the following four steps.

**Step** 1: Domain definition and interval division. First, define the fund expenditure time series data *Y*(*t*), *t* = 1, 2, …, *T* the domain of discourse is *U* = [*y*_min_, *y*_max_], where *y*_min_ = min *Y*(*t*), *y*_max_ = max *Y*(*t*). Evenly divide *U* into *K* fund expenditure intervals *u*_*i*_, *i* = 1, 2, …, *K*, then *U* can be expressed as *U* = {*u*_1_, *u*_2_, …, *u*_*K*_}.
**Step** 2: Define a fuzzy set (linguistic value) for the fund expenditure. Based on the divided fund expenditure interval, *K* fuzzy sets can be defined, which are defined as *E*_1_, *E*_2_, …, *E*_*K*_, which can be specifically expressed as,
$$\left\{\begin{array}{c} E_{1}=\frac{f_{E_{1}}(u_{1})}{u_{1}}+\frac{f_{E_{1}}(u_{2})}{u_{2}}+\cdots +\frac{f_{E_{1}}(u_{K})}{u_{K}}\\ E_{2}=\frac{f_{E_{2}}(u_{1})}{u_{1}}+\frac{f_{E_{2}}(u_{2})}{u_{2}}+\cdots +\frac{f_{E_{2}}(u_{K})}{u_{K}}\\ \ldots \\ E_{K}=\frac{f_{E_{K}}(u_{1})}{u_{1}}+\frac{f_{E_{K}}(u_{2})}{u_{2}}+\cdots +\frac{f_{E_{K}}(u_{K})}{u_{K}} \end{array}\right.$$ (4)
  where $f_{E_{i}}(u_{j})$ represents the membership degree of *u*_*j*_ belonging to the fuzzy set *E*_*i*_. The triangular fuzzy membership function commonly is used in this paper [39], which is expressed as,
$$f_{E_{i}}(u_{j})=\left\{\begin{array}{cc} 1, & i=j\\ 0.5, & i=j+1\;\text{or}\;i=j-1\\ 0, & \text{other} \end{array}\right.$$ (5)
  where *i* = 1, 2, …, *K* and *j* = 1, 2, …, *K*.3
**Step** 3: Establish a fuzzy relation group and calculate the weight matrix. Suppose the fuzzy set of fund expenditure at time *t* is *E*_*i*_, and the fuzzy set of fund expenditure at time *t*+1 is *E*_*j*_. In that case, a fuzzy relationship can be constructed, *E*_*i*_ → *E*_*i*_. For the time series data set of fund expenditure, select *N*+1 samples as training data, and build a fuzzy relationship according to the above method. A fuzzy relationship group composed of *N* fund expenditures fuzzy relationships can be obtained. The same fuzzy relationship among them is counted. Suppose the number of occurrences of the fuzzy relationships *E*_*i*_ → *E*_*j*_ is *m*_*ij*_ times (*m*_*ij*_ = 1, 2, …, *N*, *i* = 1, 2, …, *K* and *j* = 1, 2, …, *K*), and there are,
$$\sum_{i=1}^{K}\sum_{j=1}^{K}m_{ij}=N$$ (6)
  where the number of occurrences *m*_*ij*_ of fuzzy relationships that do not occurrences is 0. According to *m*_*ij*_, the fuzzy relationship matrix *R*_*M*_ can be obtained as,
$$R_{\text{M}}=\left(\begin{array}{cccc} m_{11} & m_{12} & \cdots & m_{1K}\\ m_{21} & m_{22} & \cdots & m_{2K}\\ \vdots & \vdots & \ddots & \vdots \\ m_{K1} & m_{K1} & \cdots & m_{KK} \end{array}\right)$$ (7)
  According to the fuzzy relationship matrix, the weight matrix of the fuzzy relationship can be calculated [11], which can be expressed as,
$$W_{\text{M}}=\left(\begin{array}{cccc} w_{11} & w_{12} & \cdots & w_{1K}\\ w_{21} & w_{22} & \cdots & w_{2K}\\ \vdots & \vdots & \ddots & \vdots \\ w_{K1} & w_{K2} & \cdots & w_{KK} \end{array}\right)$$ (8)
  where $w_{ij}=\frac{m_{ij}}{\sum_{k=1}^{K}r_{ik}}$, *i* = 1, 2, …, *K* and *j* = 1, 2, …, *K*.
**Step** 4: Calculate the predicted value of fund expenditure. Suppose the fund expenditure at time *q* is *Y*(*q*), and the predicted value of fund expenditure at time *q*+1 is $\hat{Y}\left(q+1\right)$, then the fund expenditure prediction model takes *Y*(*q*) as the input and $\hat{Y}\left(q+1\right)$ is the output. First, by fuzzifying *Y*(*q*) according to Steps 1 and 2, the membership degrees *v*_*i*_ of *Y*(*q*) belonging to different fuzzy sets *E*_*i*_ can be obtained, *i* = 1, 2, …, *K*. According to the weight matrix of the fuzzy relationship in Step 3, it can be known that the weights of the fuzzy relationship *E*_*i*_ → *E*_1_, *E*_*i*_ → *E*_2_, …, *E*_*i*_ → *E*_*i*_, …, *E*_*i*_ → *E*_*K*_ are *w*_*i*1_, *w*_*i*2_, …, *w*_*ij*_, …, *w*_*iK*_, then the initial predicted value of fund expenditure can be expressed as,
$${\hat{Y}}_{0}\left(q+1\right)=\sum_{i=1}^{K}f_{\text{DF}}(E_{i})w_{iK}$$ (9)
  where *f*_DF_(*E*_*i*_) is the defuzzification value of the fuzzy set *E*_*i*_ [40],
$$f_{\text{DF}}(E_{i})=\frac{\sum_{i=1}^{K}f_{E_{i}}(u_{j})Y(h)}{\sum_{i=1}^{K}f_{E_{i}}(u_{j})}$$ (10)
  where *Y*(*h*) is the observed value of fund expenditure belonging to the interval *u*_*j*_.

Due to the periodicity of the plan and arrangement of the annual fund revenue and expenditure of universities, the periodicity should be considered when predicting fund revenue and expenditure. Therefore, this paper introduces the periodic factor *λ* ∈ [0,1], then the final predicted value of fund expenditure can be expressed as,

$$\hat{Y}\left(q+1\right)=\lambda {\hat{Y}}_{0}\left(q+1\right)+(1-\lambda )Y(q+1-T_{\text{S}})$$ (11)

where *Y*(*q* + 1 − *T*_S_) represents the observed value of fund expenditure in the previous cycle time *q* + 1 − *T*_S_, where *T*_*S*_ represents the time period.

## 4 Experiments and analysis

**Fig 1:**
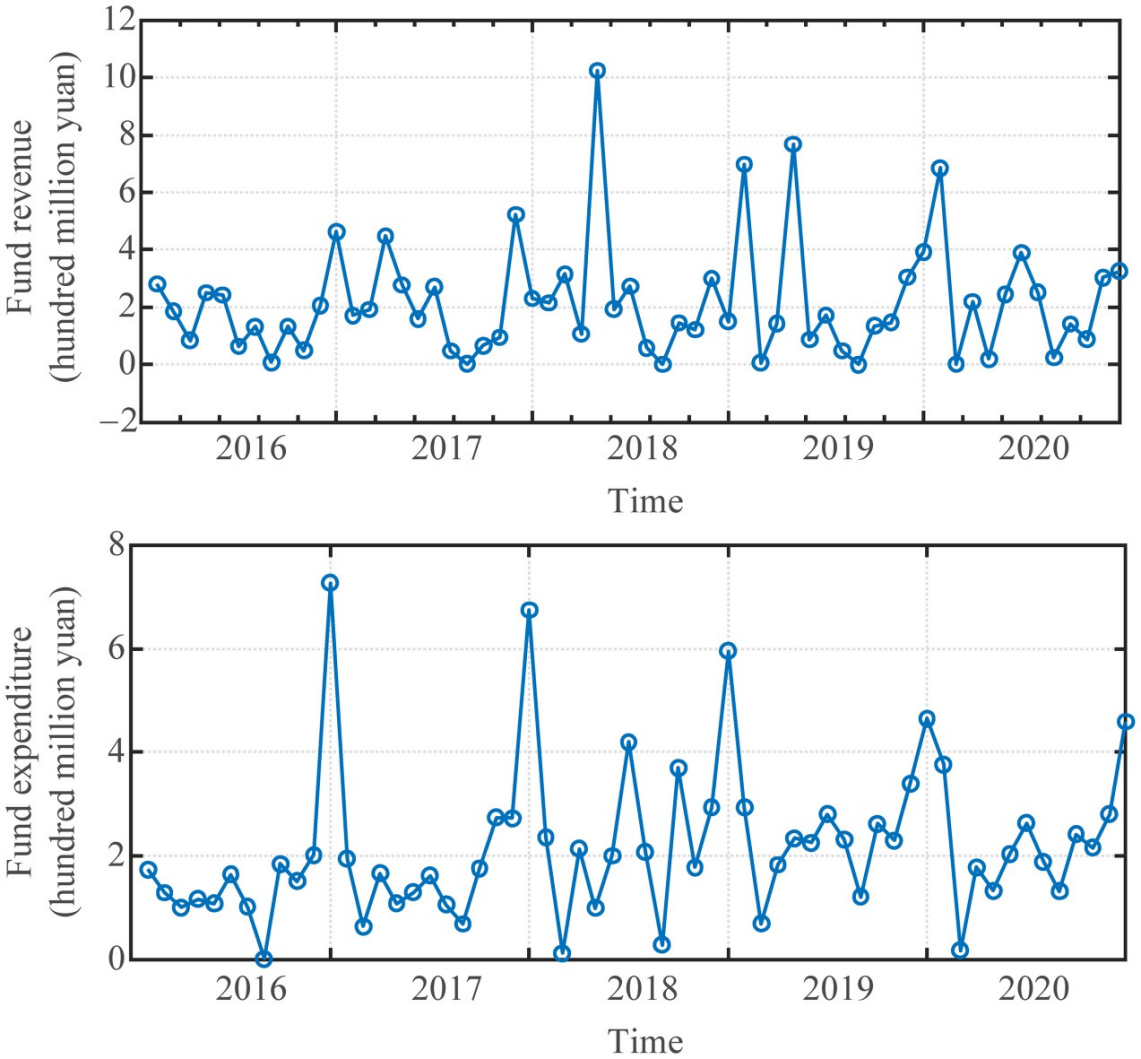
Raw data of monthly fund revenue and expenditure of a university.

The monthly fund revenue and expenditure data of a university in China from January 2016 to December 2020 are taken as a sample. The raw data of monthly fund revenue and expenditure is shown in Fig 1. The fund expenditure data clearly indicates that the data is periodicity, which is one year. The reason for the periodicity of the data is that universities have fixed opening and vacation times, and relatively fixed arrangements for courses and scientific research projects. This reason makes the fund revenue and expenditure in the university show a clear periodicity. Therefore, this paper chooses the time period *T*_*S*_ as 12 in the subsequent experiments.

**Fig 2:**
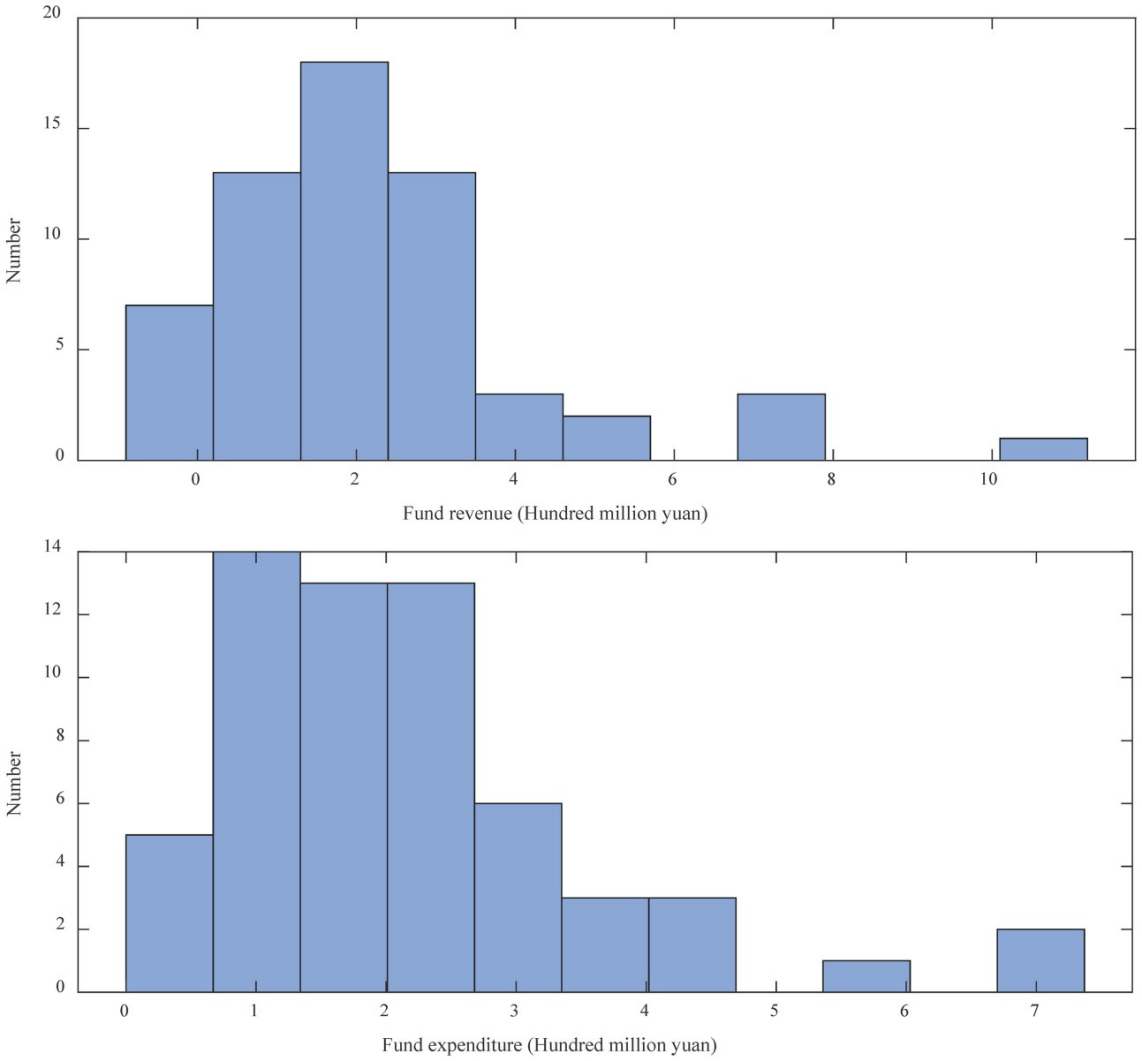
Data distribution of fund revenue and expenditure.

Descriptive statistics of fund revenue and expenditure data are given. The mean, maximum, minimum, median, sample variance, and standard deviation of fund revenue data are 2.1760, 10.2508, -0.0083, 1.7050, 3.9818, and 1.9955. Meantime, The mean, maximum, minimum, median, sample variance, and standard deviation of fund expenditure data are 2.1743, 7.2733, 0.0090, 1.9178, 2.0849, and 1.4439. Besides, the data distribution of fund revenue and expenditure are shown in Fig 2.

To verify the stationary of the data, the augmented dickey-fuller test for unit root is performed. Firstly, the null hypothesis (H0) is defined as the original data being non-stationary. The augmented dickey-fuller test unit root is used to obtain the statistic *T*_h_. The corresponding *p*-value can be obtained from Th. If the 1–*p*-value is less than the confidence level (*α* = 0.01), then H0 is rejected. That is, when the significance level is 0.001, the original data is stationary.

After calculation, for the fund revenue data, *T*_h_ = −2.6164, *p*-value = 0.0098. Therefore, when the significance level is 0.001, the fund revenue data is stationary. Meanwhile, for the fund expenditure data, *T*_h_ = −4.3194, *p*-value = 0.0010. Therefore, when the significance level is 0.001, the fund expenditure data is stationary. The stationarity of the fund revenue and expenditure data provides the basis for the data forecasts.

There are 60 samples of monthly fund revenue and expenditure data for five years. 80% of the data is used for training the fuzzy time series-based prediction model of fund revenue and expenditure, and 20% of the data is used for prediction model validation. This paper adopts the root mean square error (RMSE), the maximum absolute error (MaxAE), and the average relative error (ARE) [41] as an evaluation index to verify the effect of the fund revenue and expenditure prediction model.

$$\text{RMSE}=\sqrt{\frac{1}{T}\sum_{t=1}^{T}(\hat{Y}(t)-Y(t){)}^{2}}$$ (12)

$$\text{MaxAE}=\text{max}|\hat{Y}(t)-Y(t)|$$ (13)

$$\text{ARE}=\frac{1}{T}\sum_{t=1}^{T}\frac{\left|\hat{Y}(t)-Y(t)\right|}{Y(t)}$$ (14)

where *T* represents the number of verification samples, $\hat{Y}\left(t\right)$ represents the predicted value of the und revenue and expenditure prediction model, and *Y*(*t*) represents the actual value of the fund revenue and expenditure.

In the fund revenue and expenditure prediction models, only the number of fund revenue or expenditure intervals *K* and the periodic factor *λ* need to be discussed and determined. Since the RMSE indicator is a comprehensive measure of the error, it can better evaluate the prediction effect of the fund revenue and expenditure prediction models. Therefore, the RMSE indicator is mainly used as the primary reference in the parameter discussion.

**Fig 3:**
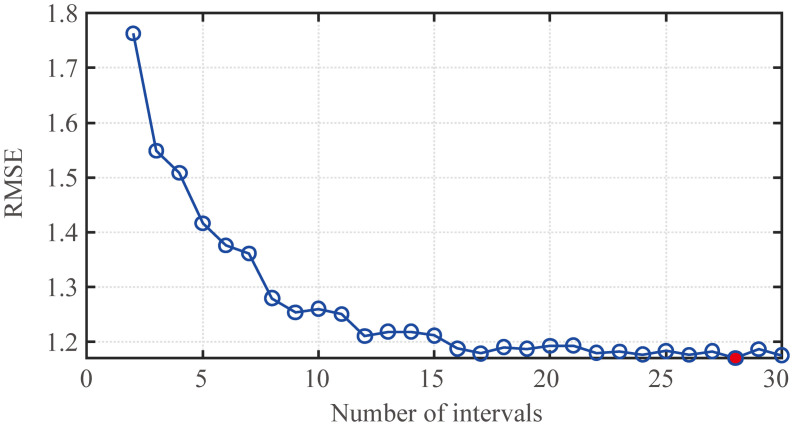
Effect of fund revenue prediction model under different numbers of intervals (*λ* = 0.4).

**Fig 4:**
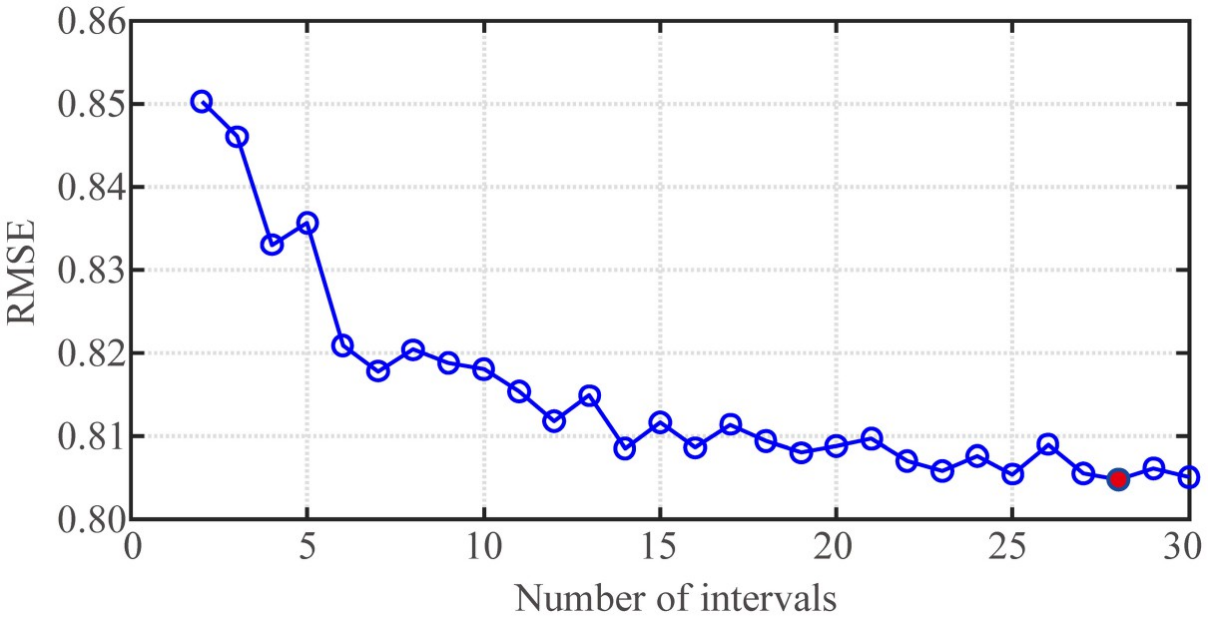
Effect of fund expenditure prediction model under different numbers of intervals (*λ* = 0.1).

First, when the periodic factor *λ* is fixed, the prediction effect of the fund revenue and expenditure prediction models under different numbers of intervals *K* is discussed. The prediction effect of the fund revenue prediction model under different numbers of intervals is shown in Fig 3. The prediction effect of the fund expenditure prediction model is shown in Fig 4. The indicator in Figs 3 and 4 is the RMSE of the training models, and the number of intervals *K* is selected from 2 to 30. From the figure that the RMSE shows a decreasing trend with the increase of *K*, but when *K* = 28, the fund revenue prediction model, and the fund expenditure prediction model both reach the minimum RMSE. Therefore, this paper considers that both models achieve the best prediction performance currently.

**Fig 5:**
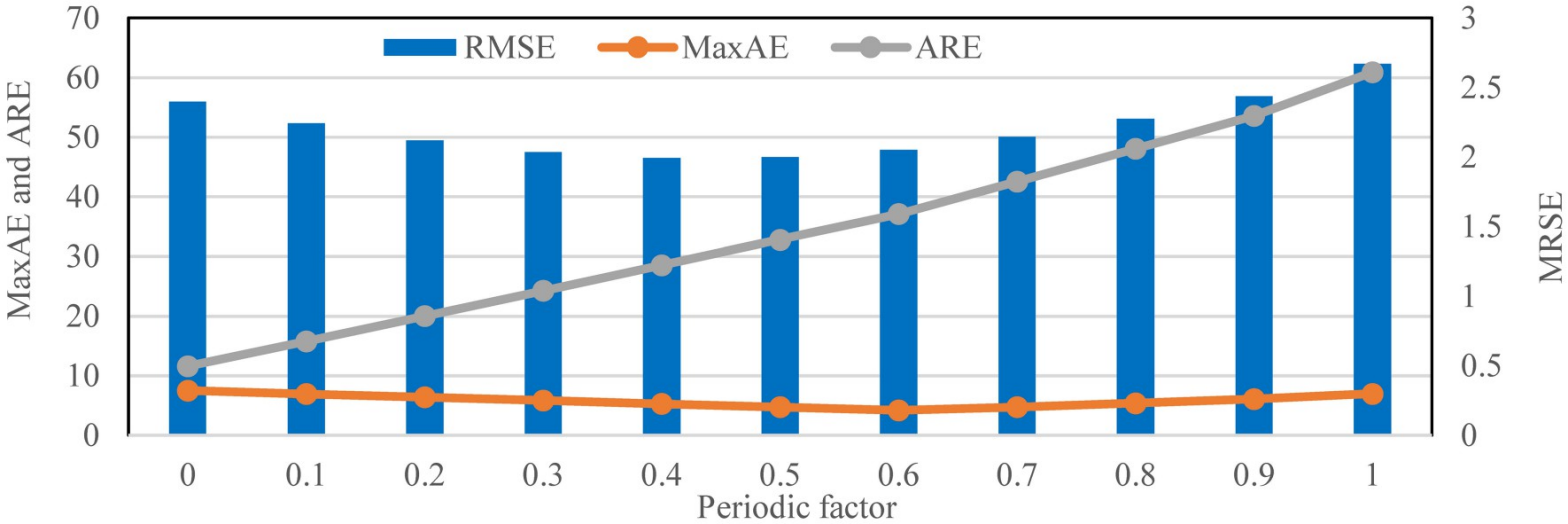
Indicator changes of fund revenue prediction model under different periodic factors (*K* = 28).

**Fig 6:**
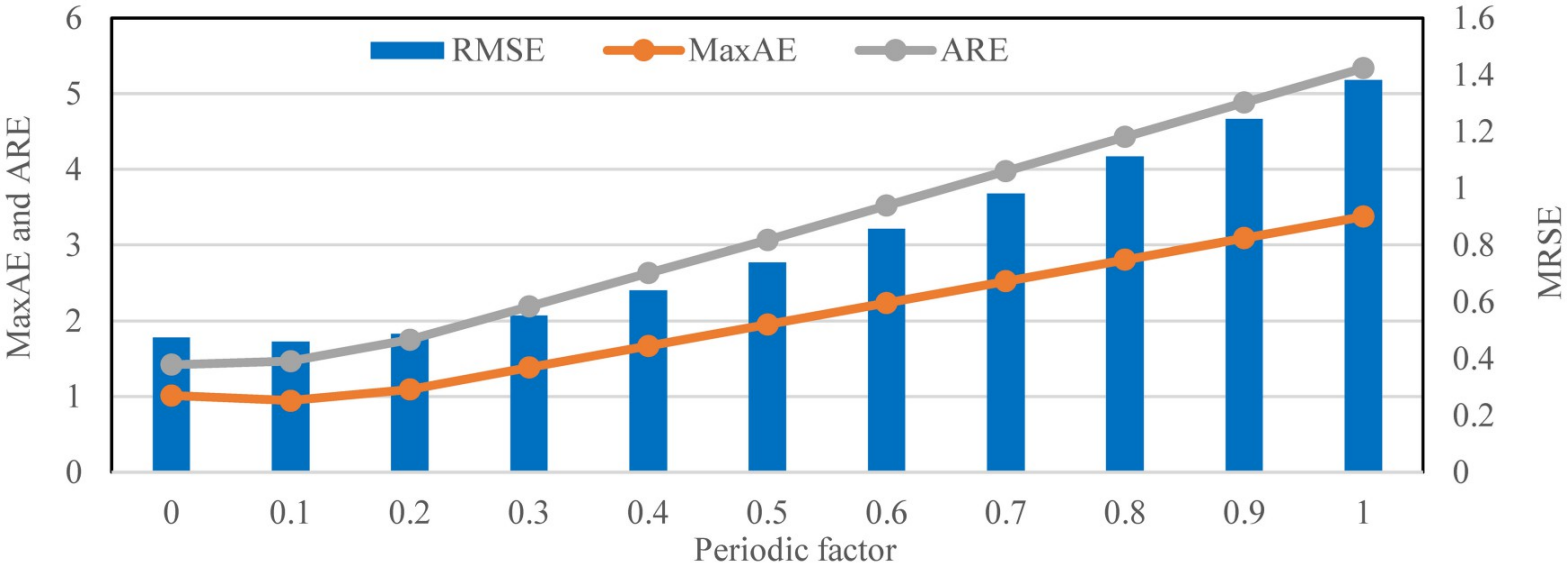
Indicator changes of fund expenditure prediction model under different periodic factors (*K* = 28).

After the interval number *K* = 28 of the fund revenue and expenditure prediction models is determined, it is necessary to discuss the role and selection of the periodic factor *λ*. This paper uses the uniform step test method to test all model effects from *λ* = 0 to *λ* = 1, and the step value is 0.1. The RMSE, MaxAE, and ARE of the fund revenue prediction model and fund expenditure prediction model under different periodic factors λ are shown in Table 1, and their changes are shown in Figs 5 and 6. It is worth noting that when *λ* = 0, the current fund revenue or expenditure prediction model is only related to the observation value of the previous periodic. When *λ* = 1, it means that the current fund revenue or expenditure prediction model is only associated with the output value of the fuzzy time series.

**Table 1:** Indicators of the fund revenue and expenditure prediction models under different periodic factors (*K* = 28).

Fund revenue prediction model	*λ*	0	0.1	0.2	0.3	0.4	0.5	0.6	0.7	0.8	0.9	1
	RMSE	2.398	2.242	2.119	2.035	**1.994**	1.999	2.050	2.144	2.274	2.436	2.671
	MaxAE	7.493	6.939	6.385	5.832	5.278	4.724	**4.171**	4.746	5.417	6.088	6.967
	ARE	**4.128**	8.803	13.611	18.442	23.272	28.102	32.935	37.791	42.649	47.508	53.959
Fund expenditure prediction model	*λ*	0	0.1	0.2	0.3	0.4	0.5	0.6	0.7	0.8	0.9	1
	RMSE	0.474	**0.460**	0.488	0.552	0.641	0.739	0.857	0.982	1.112	1.245	1.381
	MaxAE	1.011	**0.944**	1.095	1.381	1.666	1.951	2.236	2.522	2.807	3.092	3.377
	ARE	**0.407**	0.523	0.655	0.809	0.964	1.116	1.285	1.453	1.622	1.791	1.959

From the table, in the fund revenue prediction model, the change of *λ* has an essential influence on the prediction effect of the model. The RMSE indicator achieves the minimum value when *λ* = 0.4 and shows growth on both sides of this value, so in the current situation, *λ* = 0.4 is the optimal parameter. However, the MaxAE indicator achieves the minimum value when *λ* = 0.6, while the ARE indicator achieves the minimum when *λ* = 0. When multiple indicators are evaluated, it is not easy to determine the best one. This paper has made it clear that the RMSE indicator is the primary reference. Therefore, the optimal adjustment factor of the capital income prediction model is *λ* = 0.4.

Besides, in the fund expenditure prediction model, the influence of the change of *λ* on the prediction effect of the model is mainly concentrated in the front end. The RMSE indicator achieves the minimum value when *λ* = 0.1, and the MaxAE indicator also reaches the minimum value. So *λ* = 0.1 is the best choice for the optimal two indicators, but the ARE indicator still achieves the minimum value when *λ* = 0. Therefore, through overall consideration, the optimal periodic factor of the fund expenditure prediction model is *λ* = 0.1.

**Fig 7:**
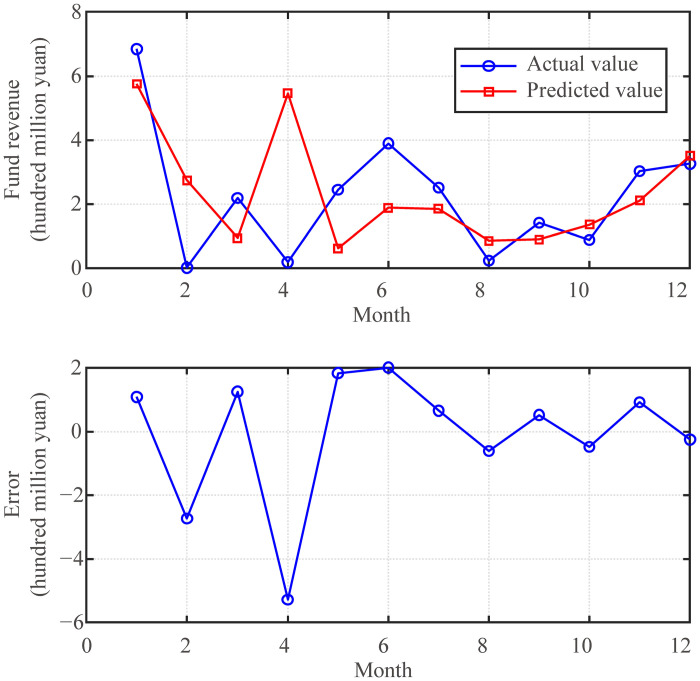
Effect of funds revenue prediction model (*K* = 28, *λ* = 0.4).

The prediction effect of the fund revenue prediction model with the optimal parameters *K* = 28 and *λ* = 0.4 are shown in Fig 7. From the figure, the fund revenue prediction model has maintained a good trend forecasting effect overall, but in April 2020, this data showed an abnormal phenomenon. The main reason for this phenomenon is that it was a special January after the outbreak of the COVID-19 pandemic, just in time for the start of universities. As can be seen from the data in Fig 1, there would be a peak in fund revenue this month in previous years and a trough in April 2020. This is due to the great changes in external conditions, so the fund revenue prediction model cannot adapt to this sudden change.

**Fig 8:**
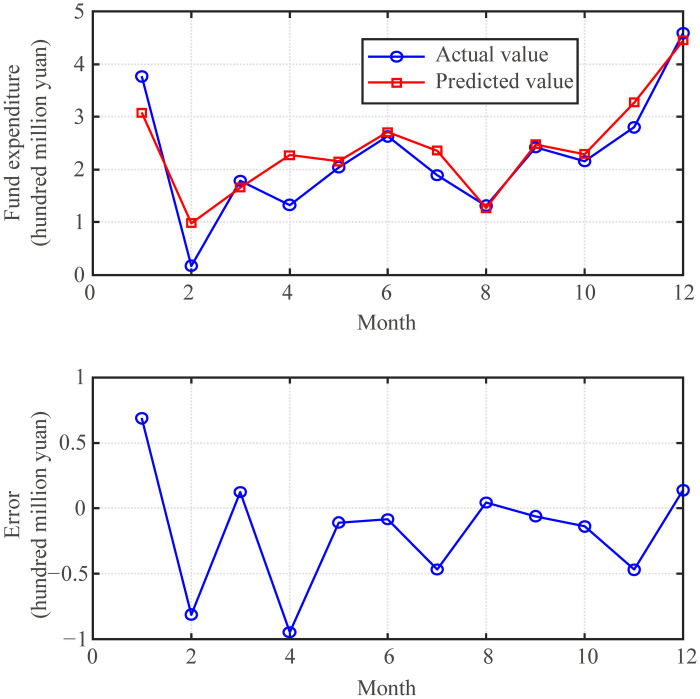
Effect of funds expenditure prediction model (*K* = 28, *λ* = 0.1).

The prediction effect of the fund expenditure prediction model with the optimal parameters *K* = 28 and *λ* = 0.1 are shown in Fig 8. From the figure, the trend prediction effect of the fund expenditure prediction model maintains a good trend prediction effect and predicts very accurately in terms of specific values. It was also affected by the COVID-19 pandemic, but the prediction effect of the fund expenditure prediction model did not appear to be particularly abnormal. This is because the expenditures of universities can be operated following fixed procedures, and the online processing of fund expenditures maintains regular changes.

**Fig 9:**
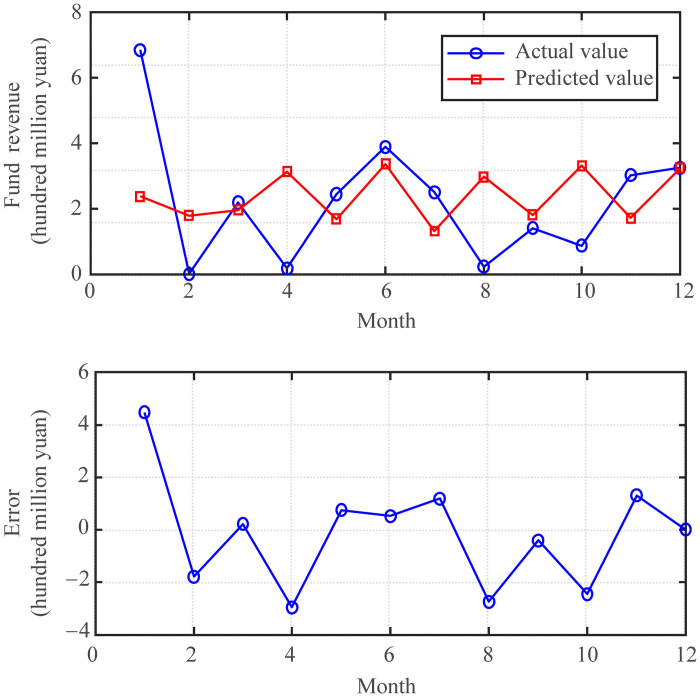
Prediction effect of fund revenue by ARMA model.

**Fig 10:**
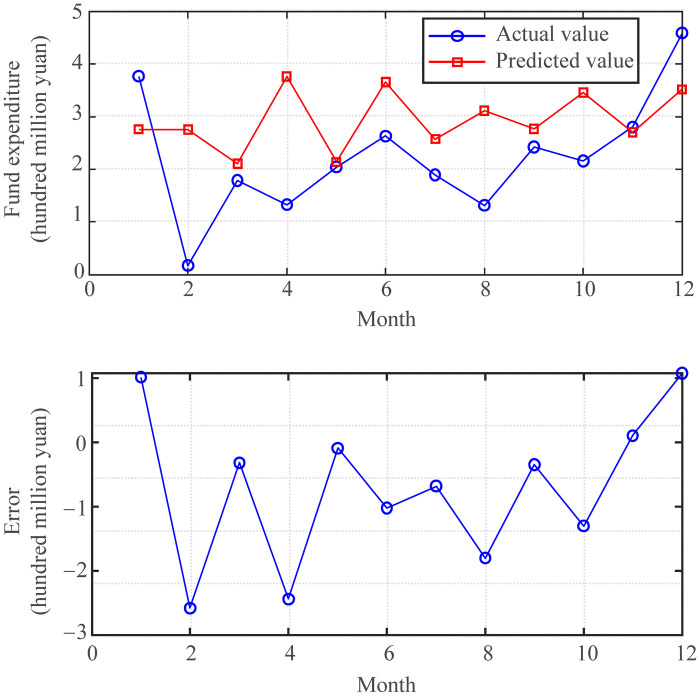
Prediction effect of fund expenditure by ARMA model.

To verify the progressiveness of our model, we compared the Autoregressive moving average model (ARMA) model. An autoregressive model of order 2 and a moving average model of order 2 were determined. The prediction effect of fund revenue by ARMA model is shown in Fig 9, and the prediction effect of fund expenditure by ARMA model is shown in Fig 10. By calculating, the ARMA modelof fund revenue can be obtained as MSE = 2.0319, MaxAE = 4.4693, and ARE = 16.0149. Also, the ARMA model of fund expenditure can be obtained as RMSE = 1.338, MaxAE = 2.5808, and ARE = 1.695. From the Table 1, it is clear that our model outperforms the ARMA model.

By discussing the parameters of the fund revenue and expenditure prediction models and the prediction effect of the model, it can be found that the proposed prediction model in this paper is effective. It not only considers the periodicity of the fund revenue and expenditure of universities but also considers the time series from the perspective of the time series itself. So, the fund revenue and expenditure prediction models have a good prediction effect, providing a powerful reference for financial management and decision-making in universities. To facilitate the validation of our model, the code for the model is provided on the webpage: https://doi.org/10.7910/DVN/WKOHGI. The experimental results in this paper are obtained from several experiments to ensure the robustness of the model.

## 5. Conclusions

This paper presents a prediction model of university fund revenue and expenditure based on fuzzy time series with a periodic factor to solve the problem of the lack of solid data reference for financial management and decision-making in universities. On this basis of fuzzy time series, the periodic factor of university funds is introduced, and the periodic factor is used to realize the adjustment of the predicted value of the fuzzy time series and the periodic observation value. Experiments are carried out with the actual financial data of a university in China. The experimental results show the effectiveness of the proposed model, which can provide a powerful reference for financial management and decision-making in universities.

This method used in selecting periodic factors for the proposed fund revenue and expenditure prediction model in this paper cannot exhaustively list all options. The prediction effect still has room for improvement. Besides, university fund revenue and expenditure periodicity are essential for prediction. Therefore, we will further develop the parameter selection and periodic utilization in the fund revenue and expenditure prediction model in the future.
